# The EMT-activator ZEB1 induces bone metastasis associated genes including BMP-inhibitors

**DOI:** 10.18632/oncotarget.3882

**Published:** 2015-05-05

**Authors:** Kerstin Mock, Bogdan-Tiberius Preca, Tilman Brummer, Simone Brabletz, Marc P. Stemmler, Thomas Brabletz

**Affiliations:** ^1^ Department of Visceral Surgery, University Medical Center Freiburg, Freiburg, Germany; ^2^ Faculty of Biology, University of Freiburg, Freiburg, Germany; ^3^ Institute of Molecular Medicine and Cell Research, University Medical Center Freiburg, Germany and Center for Biological Signalling Studies *BIOSS*, Albert-Ludwigs-University Freiburg, Freiburg, Germany; ^4^ Department of Experimental Medicine I, Nikolaus-Fiebiger-Center for Molecular Medicine, University Erlangen-Nürnberg, Erlangen, Germany

**Keywords:** breast cancer, bone metastasis, epithelial-mesenchymal transition (EMT), BMP-inhibitor, ZEB1

## Abstract

Tumor cell invasion, dissemination and metastasis is triggered by an aberrant activation of epithelial-to-mesenchymal transition (EMT), often mediated by the transcription factor ZEB1. Disseminating tumor cells must acquire specific features that allow them to colonize at different organ sites. Here we identify a set of genes that is highly expressed in breast cancer bone metastasis and activated by ZEB1. This gene set includes various secreted factors, e.g. the BMP-inhibitor FST, that are described to reorganize the bone microenvironment. By inactivating BMP-signaling, BMP-inhibitors are well-known to induce osteolysis in development and disease. We here demonstrate that the expression of ZEB1 and BMP-inhibitors is correlated with bone metastasis, but not with brain or lung metastasis of breast cancer patients. In addition, we show that this correlated expression pattern is causally linked, as ZEB1 induces the expression of the BMP-inhibitors NOG, FST and CHRDL1 both by directly increasing their gene transcription, as well as by indirectly suppressing their reduction via miR-200 family members. Consequently, ZEB1 stimulates BMP-inhibitor mediated osteoclast differentiation. These findings suggest that ZEB1 is not only driving EMT, but also contributes to the formation of osteolytic bone metastases in breast cancer.

## INTRODUCTION

Breast cancer is the second leading cause of cancer related death in women and more than 70% of breast cancer patients in the advanced stage develop bone metastases predominantly with an osteolytic phenotype [1–3]. It is widely accepted that the initiation of the multistep metastatic process is enhanced by an aberrant activation of the embryonic epithelial-to-mesenchymal transition (EMT) program, which enables the cells to disseminate from the primary tumor and to invade into the surrounding tissue. The EMT program is induced by stromal derived factors like transforming growth factor β (TGFβ) and converges in the activation of so-called EMT-inducers, particularly of the Snail- and ZEB-family (zinc finger E-box binding homeobox; ZEB1 and ZEB2) in the tumor cells [4]. This group of transcription factors is predominantly described to repress epithelial gene expression by binding to E-boxes in their promoter regions [5, 6]. However, on some genes, e.g. α smooth muscle actin (αSMA), the EMT-inducers have opposite effects and activate transcription, which seems to be dependent on the recruitment of different co-factors [7–9]. The EMT process has also been linked to the acquisition of stemness properties [10–12]. We and others have shown that the double-negative feedback loop between the EMT-inducer ZEB1 and the stemness suppressing members of the miRNA-200 family (miRs-141, -200a, -200b, -200c, -429) plays an important role in regulating EMT and the subsequent metastatic process [11, 13, 14]. In breast cancer, high expression of the EMT-inducer ZEB1 is strongly correlated with the estrogen receptor (ER) negative claudin-low subtype, which shows an intrinsic EMT phenotype, whereas its expression is very low in ER positive breast cancer cases [15, 16].

Besides invasive properties, cancer cells need to acquire features that allow them to settle and colonize at metastatic organ sites. Diverse gene signatures for organ-specific metastasis of breast cancer were described in the last decade [17–19]. Kang et al defined a bone metastasis gene signature from bone metastases of orthotopic mouse xenografts. This signature represents genes involved in late events of the bone metastatic process, including many secreted factors and cell surface proteins, described to manipulate the bone microenvironment, e.g. matrix metalloproteinase 1 (MMP1) [17]. Subsequent stimulation of bone resorbing osteoclasts or inhibition of bone forming osteoblasts results in the degradation of the bone and release of bone matrix-embedded tumor-promoting growth factors such as TGFβ. This suggests the action of a vicious cycle between the growth of the metastatic tumor mass and bone resorption [2, 20]. Recently, the EMT-inducer ZEB1 was not only described as initiator of metastasis, but also to induce osteoclast formation and to inhibit osteoblast differentiation by regulating MMP1 expression in an *in vitro* system of breast cancer bone metastasis [21].

Bone morphogenetic proteins (BMPs) are multifunctional growth factors that belong to the TGFβ superfamily [22]. They were initially identified by their ability to induce ectopic bone formation and are now known for their important role in morphogenesis during development [23–25]. Besides stimulating bone formation BMPs are able to induce differentiation of stem cells, e.g. in the intestinal epithelium [26, 27]. The activity of the BMP signaling pathway is modulated by BMP-inhibitors, e.g. Noggin (NOG), Follistatin (FST) and Chordin-like 1 (CHRDL1). These proteins are secreted to the extracellular space where they competitively bind to BMPs and thus antagonize their function [28]. Consequently, transgenic mice overexpressing the BMP-inhibitor Nog under the control of the osteocalcin promoter were shown to suffer from osteopenia and reduced bone formation [29]. Recently, NOG was also described to facilitate bone colonization of metastatic breast cancer cells. NOG upregulation in breast cancer cells contributes to the initiation of metastasis formation by stimulating stemness properties. At the same time tumor cell secreted NOG induces osteoclast differentiation and subsequent bone degradation at the metastatic site [30].

Here we show that the EMT-inducer ZEB1 activates the expression of genes, previously associated with breast cancer bone metastasis, including the BMP-inhibitors NOG, FST and CHRDL1. These data indicate ZEB1 as a crucial mediator of the bone metastatic process.

## RESULTS

### The expression of *ZEB1* and BMP-inhibitors correlates with breast cancer bone metastasis

**Figure 1:**
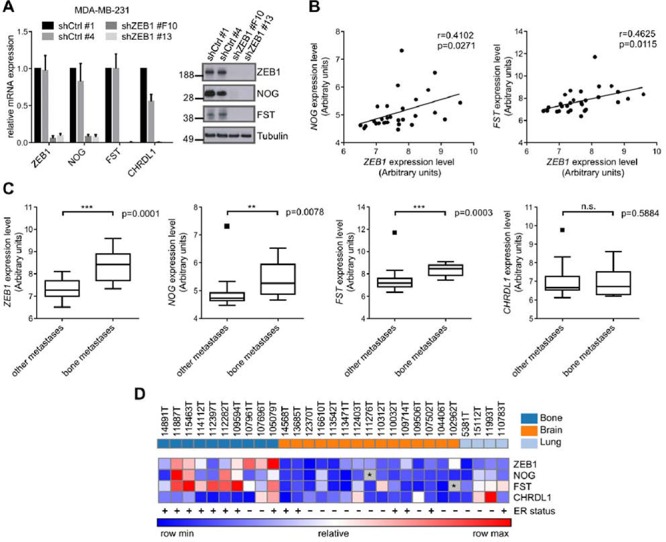
The expression of ZEB1 and BMP-inhibitors correlates with breast cancer bone metastasis **A.** Stable shRNA mediated knockdown of ZEB1 in MDA-MB-231 results in downregulation of the BMP-inhibitors NOG, FST and CHRDL1 relative to control clone shCtrl #1 on mRNA and protein level, measured by qRT-PCR and western blot analysis. qRT-PCR data are represented as mean +SD. **B.** The expression of *ZEB1* and *NOG* (left), as well as *ZEB1* and *FST* (right) is significantly correlated in metastatic samples from bone, lung and brain of breast cancer patients (GSE14020). **C.** Comparison of mRNA expression levels of bone metastases to other metastatic sites in a set of samples from bone, lung and brain metastases of breast cancer patients (GSE14020) reveals higher expression of *ZEB1*, *NOG* and *FST* in bone metastases. Boxplots are depicted according to Tukey. **D.** Heatmap showing the correlated expression of *ZEB1*, *NOG* and *FST* in metastatic samples (GSE14020). The ER status is depicted with + or −. The outlier values for *FST* and *NOG* that were observed in the boxplots in C were excluded and depicted as grey boxes with *. The sites of metastases, bone, brain and lung, are indicated in the top row in dark blue, orange and light blue, respectively.

The transcription factor ZEB1 predominantly acts as transcriptional repressor, e.g. of E-cadherin or the members of the miR-200 family [13]. However, when performing microarray analysis in MDA-MB-231 breast cancer cells after stable shRNA mediated knockdown of ZEB1 (shZEB1), we observed many mRNAs to be downregulated relative to control (shCtrl) (Table S1, column 5, ArrayExpress E-MTAB-3482). Among the 350 most decreased mRNAs upon ZEB1 depletion we identified the BMP-inhibitors *NOG*, *FST* and *CHRDL1*, which was confirmed by qRT-PCR and western blot (Fig. 1A, Fig. S1A).

The BMP-inhibitors NOG and FST were described to be involved in breast cancer bone metastasis [17, 30]. Although overall *NOG* levels in the primary tumor do not correlate with metastatic tropism, bone metastases express much higher levels of *NOG* than lung and brain metastases [30]. Given this observation, we checked a dataset of breast cancer metastatic samples available online (GSE14020) for expression of *ZEB1* and BMP-inhibitors. We observed significant positive correlations of *ZEB1* expression with *NOG* and *FST* expression throughout all metastatic samples (Fig. 1B), as well as elevated expression of *ZEB1* and the BMP-inhibitors *NOG* and *FST* in bone metastases, compared to lung and brain metastases (Fig. 1C, 1D). This seemed to be independent of the ER status of the metastatic tumor cells, as the dataset included ER positive and negative samples from all metastatic sites (Fig. 1D). The numbers of ER positive and negative cases reflect/reflected the fact that ER positive breast tumors predominantly metastasize to the bone, whereas ER negative tumors are more likely to form visceral and brain metastases [31, 32].

**Figure 2:**
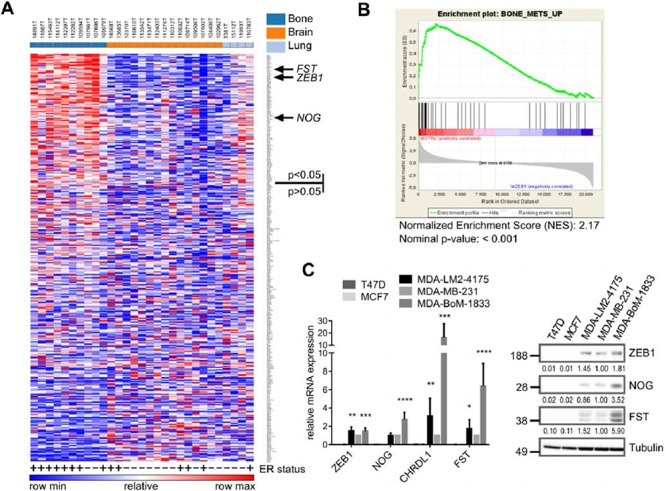
Genes positively regulated by ZEB1 are upregulated in breast cancer bone metastases **A.** Heatmap showing the differential expression of the top 350 ZEB1 target genes, determined from the MDA-MB-231 shCtrl vs. shZEB1 microarray, between bone metastases and other metastatic sites in a set of samples from bone, lung and brain metastases of breast cancer patients (GSE14020). The ER status is depicted with + or −. Genes were ranked according to differential expression in bone metastases vs. other metastatic sites by GENE-E program. 110 out of 350 genes are significantly higher expressed in bone metastases with *p* < 0.05. The sites of metastases, bone, brain and lung, are indicated in the top row in dark blue, orange and light blue, respectively. **B.** Gene-Set-Enrichment-Analysis reveals the gene set BONE_METS_UP (adapted from Kang 2003) as enriched in the shCtrl phenotype, which indicates the included genes to be downregulated after stable knockdown of ZEB1 in MDA-MB-231. **C.** The bone metastatic cell line MDA-BoM-1833 shows highest expression of BMP-inhibitors and ZEB1 when compared to parental MDA-MB-231, lung metastatic MDA-LM2–4175 and luminal breast cancer cell lines T47D and MCF7, analyzed by qRT-PCR and western blot. qRT-PCR data are represented as mean +SD.

In order to analyze whether in addition to BMP-inhibitors also other genes that are positively regulated by ZEB1 might be enriched in bone metastatic samples, we checked the top 350 genes downregulated after depletion of ZEB1 in MDA-MB-231 for their expression in the breast cancer metastases dataset. 110 out of 350 potential ZEB1 target genes were significantly increased in bone metastases compared to other metastatic sites (Fig. 2A, Table S1).

In 2003, Kang et al described a specific gene signature of up- and downregulated genes in bone metastases of breast cancer [17]. When performing a gene set enrichment analysis (GSEA) with our microarray data from MDA-MB-231 ZEB1 knockdown clones, we found Kang's gene set of upregulated genes in bone metastases to be strongly enriched in the shCtrl phenotype, representing genes positively regulated by ZEB1 (Fig. 2B). This implies that many of the genes specifically increased in bone metastases of breast cancer, e.g. the BMP-inhibitor *FST*, are dependent on ZEB1 mediated activation. Interestingly, another bone metastasis gene set, described by Smid et al 2006, was not enriched with genes positively regulated by ZEB1 (Fig. S1B) [33]. This signature was established from primary tumor material with known site of metastatic relapse and therefore describes genes responsible for early steps of bone tropism. In contrast, Kang's signature which was developed by using subclones of the triple-negative breast cancer cell line MDA-MB-231, selected for specific bone metastasis in mouse xenografts, represents later steps of bone tropism. We used one of those bone-tropic cell lines, MDA-BoM-1833, in comparison to MDA-MB-231 parental cells and the lung metastatic subclone MDA-LM2–4175, to confirm the correlated and elevated expression of ZEB1 and BMP-inhibitors detected in bone metastases also in an *in vitro* model, and to further investigate the mechanism of BMP-inhibitor activation by ZEB1.

Consistent with the *in silico* results, we found highest expression levels of ZEB1 and BMP-inhibitors in the bone metastatic cell line MDA-BoM-1833 compared to the parental MDA-MB-231 and the lung metastatic MDA-LM2–4175, as well as to the luminal, ER positive breast cancer cell lines MCF7 and T47D, which expressed neither ZEB1 nor BMP-inhibitors (Fig. 2C).

In summary, therefore the EMT-inducer ZEB1 positively regulates a set of genes, including BMP-inhibitors, with known functions during the formation of bone metastases.

### The expression of BMP-inhibitors is directly induced by ZEB1

**Figure 3:**
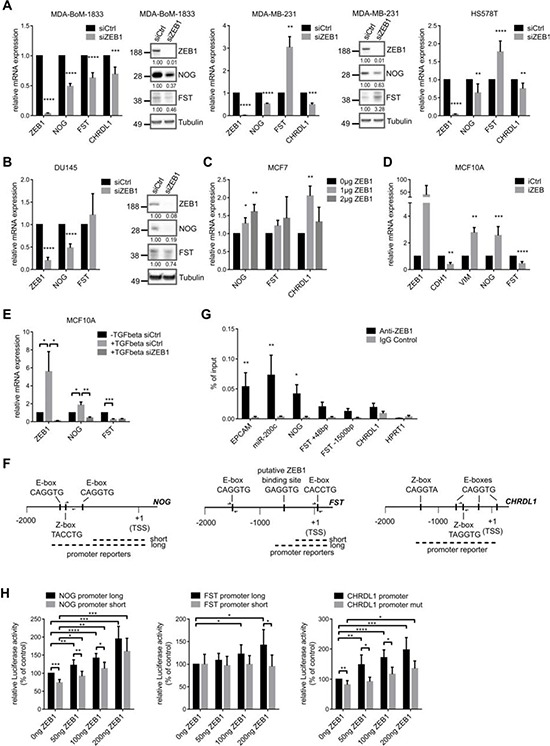
The expression of BMP-inhibitors is directly induced by ZEB1 **A.** siRNA mediated knockdown of ZEB1 in MDA-BoM-1833, MDA-MB-231 and HS578T breast cancer cells results in decreased mRNA and protein expression of the BMP-inhibitors NOG, FST and CHRDL1 relative to siCtrl, as analyzed by qRT-PCR and western blot. In MDA-MB-231 and HS578T FST is regulated antagonistically to NOG. qRT-PCR data are represented as mean +SD. **B.** siRNA mediated knockdown of ZEB1 in DU145 prostate cancer cells results in decreased mRNA and protein expression of the BMP-inhibitors NOG and FST relative to siCtrl, as analyzed by qRT-PCR and western blot. qRT-PCR data are represented as mean +SD. **C.** qRT-PCR after transfection of different concentrations of ZEB1 overexpression plasmid in MCF7 cells reveals increased mRNA levels of *NOG*, *FST* and *CHRDL1*. qRT-PCR data are represented as mean +SD. **D.** MCF10A cells with inducible ZEB1 overexpression show increased mRNA expression of *NOG*, but *FST* expression is decreased as measured by qRT-PCR. Stable iCtrl and iZEB1 cells were treated with 1 μg/ml doxycycline for 6 days to induce ZEB1 expression. qRT-PCR data are represented as mean +SD. **E.** Induction of EMT in MCF10A cells by TGFβ treatment reveals upregulation of *NOG*, which could be reversed by siRNA mediated knockdown of ZEB1. *FST* is again regulated antagonistically. qRT-PCR data are represented as mean +SD. **F.** Schematic representation of the putative promoters of human *NOG-*, *FST-* and *CHRDL1-*genes on chromosomes 17q22, 5q11.2 and Xq23, respectively. The sequence-predicted ZEB1 binding sites (E- and Z-boxes, black boxes), the regions amplified after chromatin immunoprecipitation (ChIP) (half arrows represent primer pairs) and the location of the promoter reporter constructs are indicated. Numbers are in bp relative to the transcription start site (TSS). **G.** ChIP of endogenous ZEB1 in MDA-MB-231 cells shows direct binding of ZEB1 to the promoter of *NOG*, but with less efficiency to *FST* and *CHRDL1*. Promoters of *EPCAM* and *miR-200c* are used as positive controls, *HPRT1* is used as negative control. qPCR data are represented as mean +SEM. **H.** Luciferase-reporter assays with pGL4.10-*NOG* promoter long, pGL4.10-*FST* promoter long and pGL3basic-*CHRDL1* promoter reporters reveal dose dependent increase in luciferase activity after transient ZEB1 overexpression in MCF7 cells. Shorter promoter reporters of *NOG* and *FST* lacking the E-box containing regions show reduced activation by ZEB1. Similar results were obtained when E-boxes were deleted in the *CHRDL1* promoter reporter (*CHRDL1* promoter mut). Luciferase activity relative to control is represented as mean +SD.

**Figure 4:**
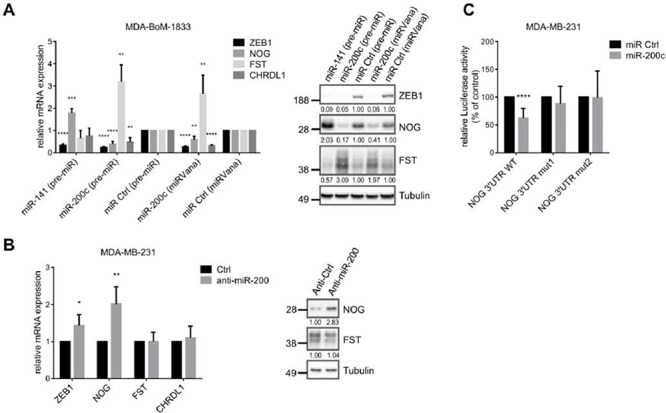
BMP-inhibitors are targets of the miR-200 family **A.** qRT-PCR and western blot analysis show targeting of ZEB1, NOG, FST and CHRDL1 by miR-200c or miR-141 (pre-miR or miRVana from life technologies) in MDA-BoM-1833. qRT-PCR data are represented as mean +SD. **B.** Inhibition of the miR-200 family with antagomiRs in MDA-MB-231 results in increased mRNA and protein expression of NOG, and slightly increased mRNA expression of *ZEB1*, analyzed by qRT-PCR and western blot. qRT-PCR data are represented as mean +SD. **C.** *NOG* 3′UTR-luciferase reporter activity is reduced after miR-200c overexpression (pre-miR) in MDA-MB-231. Sequential mutation of the miR-200c binding sites in the NOG 3′UTR prevents downregulation. Luciferase activity relative to control is represented as mean +SD.

The correlated expression of ZEB1 and BMP-inhibitors raised the question if and how the transcription factor ZEB1 is regulating the expression of BMP-inhibitors. After transient siRNA mediated knockdown of ZEB1 in MDA-BoM-1833, we observed reduced expression of the BMP-inhibitors NOG, FST and CHRDL1 (Fig. 3A). For NOG and CHRDL1 we could confirm these findings in parental MDA-MB-231 and HS578T triple negative breast cancer cell lines (Fig. 3A). However, we found FST to be increased, possibly due to an antagonistic regulatory mechanism between FST and NOG, which we also observed in other experimental settings (Figs. 3D, 3E, and 4A). This was supported by the finding that siRNA mediated depletion of either NOG or FST in different breast cancer cell lines resulted in increased expression of the other one (Fig. S2).

Depletion of ZEB1 in the prostate cancer cell line DU145, which is known to form osteolytic bone metastases, again decreased the expression of NOG mRNA and protein, as well as FST (Fig. 3B). CHRDL1 was not detectable in these cells. This result indicated that our findings hold true for different tumor entities that are able to form osteolytic bone metastases. Interestingly, we observed an upregulation of ZEB1, NOG and slightly of FST in a docetaxel resistant subclone of DU145 prostate cancer cells, supporting our data of correlated ZEB1 and BMP-inhibitor expression, here during ZEB1 mediated induction of chemoresistance [34, 35] (Fig. S1C). ZEB1 depletion in those cells could decrease NOG expression again (Fig. S1D).

Transient overexpression of ZEB1 in MCF7 breast cancer cells revealed concentration dependent upregulation of BMP-inhibitors (Fig. 3C). Likewise, in a system of doxycycline inducible ZEB1 in MCF10A cells the expression of *NOG* mRNA was increased after ZEB1 induction (Fig. 3D). However, *FST* mRNA was decreased, maybe due to the described antagonism to NOG expression. CHRDL1 was not detectable in MCF10A. Of note, these cells underwent a bona fide EMT after ZEB1 induction, as indicated by reduced E-cadherin (*CDH1*) and increased Vimentin (*VIM*) expression levels. Consistent findings were made for BMP-inhibitor expression, when EMT was induced in MCF10A cells by TGFβ treatment, resulting in increased *ZEB1* expression (Fig. 3E). The observed induction of *NOG* could subsequently be reversed by siRNA mediated ZEB1 knockdown, indicating ZEB1 as the responsible factor for *NOG* upregulation. *FST* was again decreased after EMT induction and not changed upon additional ZEB1 knockdown.

To investigate whether ZEB1 directly activates BMP-inhibitors, we analyzed the promoter regions of the BMP-inhibitor genes and detected potential ZEB1 binding sites, E-boxes or so-called Z-boxes, in all of them (Fig. 3F). By chromatin immunoprecipitation (ChIP) we found that ZEB1 was directly binding to the *NOG* promoter and with less efficiency also to *FST* and *CHRDL1* promoters (Fig. 3G). The known ZEB1 targets *miR-200c* and *EPCAM* were used as positive controls [13, 36]. In luciferase-based reporter assays we further confirmed a direct activation of the *NOG* promoter by ZEB1. ZEB1 overexpression in MCF7 led to a dose-dependent increase in luciferase activity (Fig. 3H). Similar results were detected for the promoters of *CHRDL1* and *FST*. In comparison, shorter promoter constructs of *NOG* and *FST* lacking the E-box containing regions showed significantly less or no increase in luciferase activity after ZEB1 overexpression (Fig. 3H). The same held true when E-boxes were deleted by site directed mutagenesis in the *CHRDL1* promoter reporter. In conclusion, these data demonstrated that ZEB1 induces the expression of the BMP-inhibitors NOG, FST and CHRDL1 at least partially by direct transcriptional activation.

### BMP-inhibitors are targets of the miR-200 family

In addition to a direct transcriptional activation, ZEB1 can indirectly stabilize gene expression by inhibiting the expression of microRNAs, particularly of the miR-200 family members (miRs-141, -200a, -200b, -200c, -429) [37]. We analyzed BMP-inhibitor mRNAs using online prediction tools (Targetscan, microrna.org) and detected two binding sites for miR-200c in the *NOG* 3′UTR, one binding site for miR-141 in the *FST* 3′UTR, as well as three sites for miR-200c and two sites for miR-141 in the *CHRDL1* 3′UTR. microRNA.org stated miRSVR scores <−0.1 for all binding sites, which indicate well predicted sites, and the sites are conserved at least across mammals (Fig. S3A). Accordingly, we confirmed the prediction by showing that overexpression of miR-200c or of miR-141 in the bone metastatic cell line MDA-BoM-1833 and in MDA-MB-231 leads to downregulation of NOG and CHRDL1 or of FST, respectively (Fig. 4A, Fig. S3B). The known miR-200 target ZEB1 was used as a positive control [37]. Interestingly, miR-200c overexpression increased FST expression and miR-141 overexpression increased NOG expression again suggesting a compensatory regulatory mechanism between those two BMP-inhibitors.

These results could be confirmed in DU145 prostate cancer cells and their docetaxel resistant subclone. However, the effects of miR-141 or miR-200c overexpression on BMP-inhibitors were observed predominantly on protein level, explainable by the fact that miRNAs inhibit their target genes on a posttranscriptional level and therefore not necessarily change the mRNA expression (Fig. S3C, S3D).

Inhibition of the miR-200 family with AntagomiRs led to significant upregulation of NOG, but not FST and CHRDL1 in MDA-MB-231 (Fig. 4B). A reason might be the low endogenous expression of miR-200, as well as the already high expression of ZEB1 and BMP-inhibitors in MDA-MB-231. The suppression of *NOG* by miR-200c was further investigated in a reporter assay, using a *NOG* 3′UTR luciferase construct. Overexpression of miR-200c decreased the luciferase activity and the mutation of the binding sites abolished this effect (Fig. 4C). Therefore besides directly increasing the transcription of BMP-inhibitors, ZEB1 is able to indirectly suppress their inhibition by miR-200 family members.

### BMP-inhibitors and ZEB1 stimulate the induction of osteoclast differentiation

**Figure 5:**
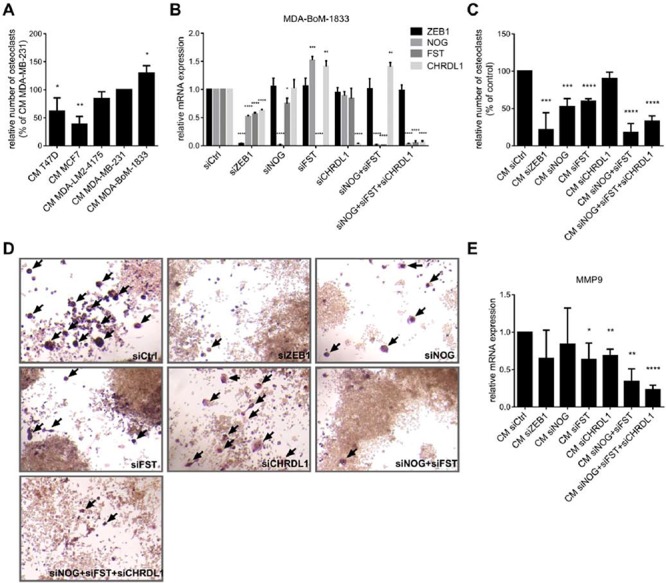
BMP-inhibitors and ZEB1 stimulate the induction of osteoclast differentiation **A.** The murine macrophage cell line Raw264.7 is treated with 5ng/ml RANKL and conditioned medium (CM) from different breast cancer cell lines to induce osteoclast differentiation. ZEB1 and BMP-inhibitor expression in the cell lines correlates with the ability of the CM to induce osteoclast differentiation. The number of osteoclasts relative to Raw264.7 treated with CM of MDA-MB-231 is represented as mean +SD. **B–D.** CM of MDA-BoM-1833 with siRNA mediated knockdown of ZEB1, NOG, FST, CHRDL1, NOG+FST and NOG+FST+CHRDL1 shows decreased ability to induce osteoclast differentiation from Raw264.7 compared to control cells. Silencing of ZEB1, NOG, FST and CHRDL1 in MDA-BoM-1833 by siRNA was proven by qRT-PCR (B). qRT-PCR data are represented as mean +SD. The number of osteoclasts relative to Raw264.7 treated with CM of siCtrl transfected MDA-BoM-1833 (represented as mean +SD) is depicted in C. Representative pictures of the osteoclasts in C are shown in D. Osteoclasts are marked by arrows. **E.** MMP9 expression in Raw264.7 after induction of osteoclast differentiation by CM of siRNA transfected MDA-BoM-1833 reflects the results of the osteoclast differentiation assay. qRT-PCR data are represented as mean +SD.

We demonstrated that elevated ZEB1 and BMP-inhibitor expression is correlated with breast cancer bone metastasis (Fig. 1). NOG expression in breast cancer cells was already described to stimulate osteoclast differentiation in osteolytic bone metastases [30]. We aimed to confirm these observations by an *in vitro* osteoclast differentiation assay. The mouse macrophage cell line Raw264.7 differentiates to osteoclasts in a RANKL-dependent manner. To test for the role of BMP-inhibitor expression by the tumor cells in facilitating this differentiation, we additionally incubated Raw264.7 cells with conditioned media (CM) from various breast cancer cells expressing different levels of ZEB1 and the BMP-inhibitors NOG and FST (Fig. 2C). The conditioned medium from bone metastatic MDA-BoM-1833, that expressed the highest levels of ZEB1 and of the BMP-inhibitors, led to the strongest osteoclast formation, compared to the parental MDA-MB-231, the lung metastatic MDA-LM2–4175 and the luminal breast cancer cell lines T47D and MCF7 (Fig. 5A). siRNA mediated knockdown of ZEB1, NOG or FST in MDA-BoM-1833 reduced the ability of the conditioned medium to induce osteoclast formation (Fig. 5B, 5C, 5D). Knockdown of CHRDL1 had no significant effect, which is consistent with the finding that CHRDL1 levels were not increased in bone metastases (Fig. 1). Interestingly, compared to ZEB1 knockdown, the individual silencing of NOG and FST in MDA-BoM-1833 inhibited the osteoclast maturation only partially. In contrast, a combination of siNOG with siFST showed a similar reduction in osteoclast formation as siZEB1, indicating that both BMP-inhibitors act in concert and are both affected by siZEB1 treatment. Moreover, the expression levels of the osteoclast gene *MMP9* in RAW264.7 cells reflected the lack of differentiation signaling cues upon NOG/FST knockdown in the tumor cells (Fig. 5E). Therefore NOG and FST seem to belong to the major secreted targets of ZEB1 that are responsible for osteoclast differentiation. Taken together, the data indicated that ZEB1 regulates the expression of secreted BMP-inhibitors in bone metastatic breast cancer cells, thereby inducing the differentiation of osteoclasts.

## DISCUSSION

In the present study we show that the EMT-inducer ZEB1 is not only driving the EMT program during the invasion process of tumor cells, but might be involved in bone metastasis formation as well. We have demonstrated that ZEB1 expression is crucial for expression of BMP-inhibitors in breast cancer cells both by directly increasing their gene transcription and by indirectly suppressing their reduction via miR-200 family members. Consequently, ZEB1 stimulates BMP-inhibitor mediated osteoclast differentiation. This relationship is further supported by the correlation of ZEB1 and BMP-inhibitor expression with bone metastasis in breast cancer patients

It is widely accepted that aberrant BMP signaling leads to impaired bone remodeling in development and disease [38]. Excess of the BMP-inhibitor NOG was recently described to be involved in the formation of osteolytic bone metastases of breast and prostate cancer, either by directly stimulating osteoclast differentiation or via reduction of osteoblast activity, thereby favoring bone resorption *in vitro* and *in vivo* [30, 39]. We here described that not only NOG but also FST and ZEB1 are overexpressed in bone metastases compared to other sites of metastasis. This seems to be independent of the ER status of the metastatic tumor cells. Even ER positive bone metastases show high expression of ZEB1 and BMP-inhibitors, although ER positive primary tumors predominantly express very low levels of endogenous ZEB1 [15, 16]. The upregulation of NOG in bone tropic compared to parental cells was already described to be independent of the ER status [30].

Furthermore, the expression of NOG and FST was directly correlated with the expression of ZEB1 across all metastatic samples, indicating a causal link in their expression pattern. ZEB1 is predominantly known for its EMT promoting activity, as well as for driving tumorigenesis and metastasis by induction of stemness properties in the tumor cells [11]. In this context, BMP-inhibitors could play an important role as well since they are well described to induce and maintain stem cell features, e.g. during development, in the intestinal crypt or during colon and breast cancer progression [26, 30, 40, 41]. In addition to its best known function as inducer of EMT, ZEB1 was shown to be required for the regulation of skeletal morphogenesis, probably by repressing osteoblast differentiation, as well as for the induction of osteoclast differentiation and thus osteolysis in breast cancer metastasis through activation of MMP1 [21, 42, 43]. In the present study we provide further evidence for the role of ZEB1 during breast cancer bone metastasis since we found a published gene set upregulated in bone metastases to be enriched with genes positively regulated by ZEB1 [17]. Due to the fact that this gene signature was defined by investigating bone tropic subclones of the MDA-MB-231 breast cancer cell line, which were isolated from experimental bone metastases, it represents genes involved during later events of the bone metastatic process, including secreted factors and cell surface proteins as candidates to mediate the interaction between the metastatic tumor cells and the bone microenvironment [17]. Thus, this gene set is not able to identify patients with increased probability of developing bone metastases from the expression profile of the primary tumor, although the bone tropic metastatic cells might already be present in the parental tumor cell population. However, they represent only a small fraction of the bulk tumor population what causes Kang's signature to be only marginally expressed in the primary tumor [44]. Therefore, ZEB1 might provide single tumor cells in the primary tumor with the ability to metastasize to the bone, where these cells are selected and enriched due to their bone metastasis favoring gene expression profile.

This seems to be reflected by our observation that ZEB1 mediated regulation of BMP-inhibitors differs between parental breast cancer cell lines and the bone metastatic MDA-BoM-1833 cells. In the bone tropic cells, NOG, FST and CHRDL1 are all positively regulated by ZEB1, whereas in different parental breast cancer cell lines only NOG and CHRDL1 are effectively targeted by ZEB1 and an antagonistic regulatory mechanism between NOG and FST seems to dominate and determine FST expression.

Furthermore, our findings might be relevant for the osteolytic bone metastasis formation of other tumor entities as well, as we could show the regulation of NOG and FST by ZEB1 in the prostate cancer cell line DU145. This cell line is known to form osteolytic bone metastases in contrast to the predominant osteogenic bone metastasis phenotype observed in prostate cancer patients [2, 45].

Various tumor-derived factors have been previously shown to be functionally involved in osteolysis by directly stimulating osteoclast differentiation, including secreted factors like RANKL, GM-CSF or PTHrP, as well as cell surface ligand jagged 1 (JAG1). The expression of JAG1 in bone-tropic breast cancer cells and the described promotion of osteolytic bone metastasis via activation of the Notch pathway in the bone microenvironment further support the role of ZEB1 in bone metastasis, as we recently showed that ZEB1 stimulates expression of JAG1 and subsequent activation of Notch-signaling through downregulation of miR-200 family members [46, 47]. Moreover, the miR-200 family member miR-429 was recently observed to be decreased in bone metastatic breast cancer cells compared to parental cells, supporting our finding of increased ZEB1 expression in bone metastases [48].

In the present study we identified different BMP-inhibitors as additional targets of the different miR-200 family members, extending the finding that NOG is a miR-200c target in ameloblast differentiation, which was published in the course of our work [49]. These data indicate that the detected molecular links are not only relevant in cancer biology but also in physiological processes.

In addition to the indirect mechanism via repression of miR-200 we identified direct activation of BMP-inhibitors by ZEB1. Although ZEB1 is mainly described as transcriptional repressor, on some genes it has the opposite effect. This seems to depend on the recruitment of different co-factors like the histone acetyltransferases p300 and PCAF, which were shown to replace the co-repressor CtBP, thereby switching ZEB1 from a repressor to an activator of transcription [7, 9]. Moreover, ZEB1 was shown to interact with SMAD1, 2 and 3, although it binds SMADs less efficiently than its homologue ZEB2. Nevertheless, ZEB1 was observed to promote the formation of a SMAD-p300 transcriptional complex by binding to p300, thereby synergizing with TGFβ and BMP induced transcriptional activation [50]. However, it still needs to be elucidated how the recruitment of different co-factors at the promoters of different ZEB1 target genes is regulated.

In summary, we suggest that aberrant increase of ZEB1 expression not only stimulates EMT-associated properties during cancer invasion and metastasis, but also leads to an upregulation of bone metastasis related genes, including BMP-inhibitors. This might be enhanced by TGFβ that is released during bone remodeling, thereby further stabilizing the EMT phenotype as well as the bone metastasis signature in the metastatic tumor cells [20].

## MATERIALS AND METHODS

### Cell culture, transfections and reporter assays

Cell lines were purchased from American Type Culture Collection (ATCC) and cultivated under standard conditions in Dulbecco's modified Eagle's medium (DMEM) supplemented with 10% fetal bovine serum except MCF10A which where cultivated in MCF10A medium (DMEM/F12 containing 5% horse serum, 20 ng/ml EGF, 0.5 μg/ml hydrocortisone, 0.1 μg/ml cholera toxin and 10 μg/ml insulin). MDA-BoM-1833 and MDA-LM2-4175 were kindly provided by Joan Massagué (Sloan-Kettering Institute for Cancer Research, New York) and Roger Gomis (Institute for Research in Biomedicine, Barcelona). DU145 and the docetaxel resistant subclone DU145 DR were kindly provided by Martin Puhr and Zoran Culig (Division of Experimental Urology, Innsbruck Medical University, Innsbruck). MCF10A cells with inducible ZEB1 expression were generated by transfecting MCF10Atet cells with linearized plasmid DNA (pTET-bsr/HAZEB1-IRES-DsRedExpress2) using Amaxa nucleofection [51]. Induction of ZEB1 expression was performed by adding 1 μg/ml doxycycline (Sigma) for six days. For induction of EMT in MCF10A cells, they were treated with 5 ng/ml TGFβ1 (PeproTech) every day until they showed a stable EMT phenotype after a minimum two weeks, before performing siRNA transfection. MDA-MB-231 stable knockdown clones for ZEB1 and control clones were described previously [52].

75 pmol miRNA (pre-miR or miRVana as indicated, Life Technologies, Ambion), 100 pmol siRNA (Silencer Select, Life Technologies, Ambion) or 200 pmol AntagomiRs (combination of anti-200a/b/c, -141 and -429 in equal amounts, Dharmacon, designed as previously described [53]) per 6-well were transfected with Lipofectamine RNAiMax transfection reagent (Life Technologies) 24 h after cell seeding. Cells were harvested after 72 h or 120 h for miRNA/AntagomiRs and siRNA respectively.

500 ng total DNA per 24-well were transfected with Fugene HD (Promega) according to the manufacturer's instructions in MCF7. Cells were harvested after 48 h.

Reporter assays were measured with Dual-Luciferase^®^ Reporter Assay System (Promega) according to the manufacturer's instructions. Firefly luciferase values were normalized against the values of a cotransfected pRL-TK Renilla-Luciferase (Promega) construct, in order to correct differences in transfection efficiencies.

Sequences of oligonucleotides, siRNAs, miRNAs and AntagomiRs are listed in Table S2.

### Plasmids

A mammalian expression vector pCI-neo-*ZEB1* encoding the full-length open reading frame of the human ZEB1 gene was kindly provided by Michel M. Sanders (University of Minnesota, Minneapolis, US). For transient ZEB1 overexpression in MCF7, 0 μg/1 μg/2 μg of pCIneo-*ZEB1* were filled with pCIneo empty vector to 2 μg of total DNA per 6well and 0 ng/50 ng/100 ng/200 ng of pCIneo-*ZEB1* were filled with pCIneo empty vector to 200 ng total DNA per 24well.

For generation of a doxycycline-inducible ZEB1 expression vector, PCR-amplified hemagglutinin (HA)-tagged ZEB1 cDNA was inserted into pMIBerry containing an internal ribosomal entry site (IRES) from the equine meningoencephalitis virus and the red fluorescent protein DsRedExpress2 [54]. The bicistronic HAZEB1-IRES-DsRedExpress2 expression cassette was further used for generation of the pTET-bsr/HAZEB1-IRES-DsRedExpress2 expression plasmid using the *Not*I site.

For construction of the *NOG* 3′UTR reporter plasmid, the full length 3′UTR was cloned downstream of the luciferase gene into the pMIR-REPORT vector (Ambion). The deletion of miRNA seed sequences was performed by site directed mutagenesis as recommended by Agilent Technologies. In Nog 3′UTR mut1 six nucleotides of the binding site around 250bp were deleted and in NOG 3′UTR mut2 six nucleotides of the site around 550bp were deleted additionally.

For construction of the promoter reporters, the nucleotides −1485 to +120 (long) and −781 to +120 (short) of *NOG* and the nucleotides −766 to +233 (long) and −471 to +233 (short) of *FST* relative to the transcription start site (TSS) were cloned into pGL4.10. The nucleotides −1439 to +3 relative to the TSS of *CHRDL1* were cloned into pGL3-basic. Deletions of the ZEB1 binding sites in the *CHRDL1* promoter reporter were performed by site directed mutagenesis as recommended by Agilent Technologies. Four nucleotides of each Z- and E-Box were deleted.

### RNA isolation and quantitative RT-PCR

RNA was isolated using RNeasy^®^ Plus Mini Kit (Qiagen). Reverse transcription of mRNA was performed using RevertAid First Strand cDNA Synthesis Kit (Thermo Scientific) according to the manufacturer's instructions. cDNA was amplified using gene-specific primers and Power SYBR Green PCR master mix (Applied Biosystems). Expression values were measured in triplicates on a Roche LightCycler 480 and normalized to human *ACTB* expression. Results are shown as the relative fold expression compared to respective control treatment.

### Immunoblotting

Cells were lysed in RIPA lysis buffer (+protease inhibitors 25xComplete, Roche). Proteins were separated on 4–12% NuPAGE^®^ Bis-Tris gels in MES-SDS running buffer and transferred to nitrocellulose membrane. For immunodetection the following antibodies were used: rabbit anti-hsZEB1 (Sigma Prestige, HPA027524; 1:5000); rat anti-hsNoggin (Regeneron, RP57–16; 1:10000); rabbit anti-hsFST (abcam, ab157471; 1:1000); mouse anti-hsTubulin (Sigma, T6199; 1:5000). Quantification of western blots was done with ImageLab software (BioRad) by normalizing specific bands to Tubulin as loading control.

### Chromatin immunoprecipitation (ChIP)

ChIP was performed as previously described, except extra crosslinking with 1.5 mM EGS for 30 min before addition of 1% formaldehyde [36]. Chromatin was incubated with anti-ZEB1 (5 μg, Santa Cruz H102, sc-25388X) and normal rabbit IgG control (5 μg, Santa Cruz, sc-2345) antibodies overnight and complexes were precipitated by protein A/G Dynabeads^®^ (Invitrogen 10002D/10004D, 25 μl each per IP). Precipitates were eluted (0.1 M NaHCO_3_, 1% SDS) and chromatin was decrosslinked by first incubating 1 h at 37°C with 250 μg/ml RNaseA and 500 μg/ml proteinase K, followed by overnight incubation at 65°C. After DNA purification the indicated regions of *NOG*, *FST* and *CHRDL1* promoters were amplified by quantitative PCR. *HPRT1* was used as negative control [13, 36].

### Osteoclast differentiation assay

For osteoclast differentiation assays Raw264.7 were treated with 5 ng/ml recombinant murine sRANK Ligand (PeproTech) and conditioned medium (CM) from different breast cancer cell lines, which were transfected with siRNA as indicated. Medium was changed every second day and tartrate-resistant acid phosphatase (TRAP) staining with the leukocyte acid phosphatase kit (Sigma-Aldrich) was performed at day six. TRAP+ cells with more than three nuclei were quantified as mature osteoclasts.

CM was collected from a defined cell number of the indicated sub-confluent tumor cells grown in DMEM with 10% FCS for 24 hours. The CM was cleared from cell debris by centrifugation at 1800 g for 10 min before diluting 1:1 with DMEM supplemented with 10% FCS and 10 ng/ml RANKL, resulting in a final concentration of 5 ng/ml RANKL. For the first treatment of Raw264.7 the CM was used freshly. The rest of the CM with RANKL was stored at −80°C until use at day 2 and 4 after initiation of osteoclast differentiation.

### Datasets and analyses

Patient data sets for different sites of metastasis were downloaded from Gene Expression Omnibus (GEO; GSE14020, GPL570, *n* = 29). This dataset includes gene expression data of metastatic samples that were microdissected (>70% tumor cell content) from frozen blocks after surgically removing the metastases. The ER status of the metastatic samples was determined from the expression data of the ER probe [32]. Probes were collapsed to the median for further analyses.

The complete expression array data set after depletion of ZEB1 in MDA-MB-231 breast cancer cells (two stable clones per group, Affymetrix GeneChip Human Genome U133 Plus 2.0 Array) was published in ArrayExpress (E-MTAB-3482). The top 350 positively regulated genes by ZEB1 were extracted from the ranked gene list (signal to noise ratio) determined by GSEA software (Broad Institute, Version 2.0.14 [55, 56]).

The Gene Set Enrichment Analysis (GSEA) was performed with GSEA software. The gene set BONE_METS_UP was defined according to Kang et al, 2003 and includes 39 genes upregulated in bone metastases [17]. This gene set was investigated together with SMID_BREAST_CANCER_RELAPSE_IN_BONE [33] and the oncogenic signature gene sets from the Molecular Signatures Database (MSigDB, Version 4.0) with default settings and 1000 gene set permutations.

Heatmaps were created with the software GENE-E (Broad Institute). For the heatmap in Fig. 1D outlier values for *FST* and *NOG* that were observed in the boxplots were excluded and depicted as grey boxes with *.

### Statistical analyses

All experiments were done at least three times. Statistical analyses of the qRT-PCR data were performed with Microsoft Excel and GraphPad Prism (GraphPad Software, Inc.). Normalized relative expression levels were used to calculate the mean and the standard deviation (SD) of all experiments. The unpaired two-tailed Student's *t*-test was used to assess statistical significances to a significance level of 95%.

Differences in gene expression in metastasis samples (GSE14020) were analyzed by the two-sided Mann-Whitney *U* test. Boxplots are depicted according to Tukey. To analyze the correlation of gene expression the Pearson correlation coefficient r was calculated. Significant enrichment of specific antibodies compared to IgG control in ChIP assays was evaluated by two-sided Mann-Whitney *U* test.

In all figures *p*-values of statistical significance are represented as follows: *p < 0.05; **p < 0.01; ***p < 0.001; ****p < 0.0001.

## SUPPLEMENTARY FIGURES AND TABLES


