# Supplementary material for: The EMT-activator ZEB1 induces bone metastasis associated genes including BMP-inhibitors
Source: Oncotarget. 2015 May 5;6(16):14399–412. doi: 10.18632/oncotarget.3882 (PMC4546475; doi:10.18632/oncotarget.3882)
Supplement: Supplementary file 1 [file oncotarget-06-14399-s001.pdf]

SUPPLEMENTARY FIGURES AND TABLES

A

	shCtrl #1	shCtrl #4	shZEB1 #F10	shZEB1 #13	Ratio
ZEB1	**1109.9**	**1003.7**	**48.9**	**76.9**	**0.1**
NOG	1723.4	1476.0	119.0	112.5	0.1
FST	2861.1	4113.0	182.3	64.6	0.0
CHRDL1	1109.9	1320.7	91.7	73.8	0.1

B

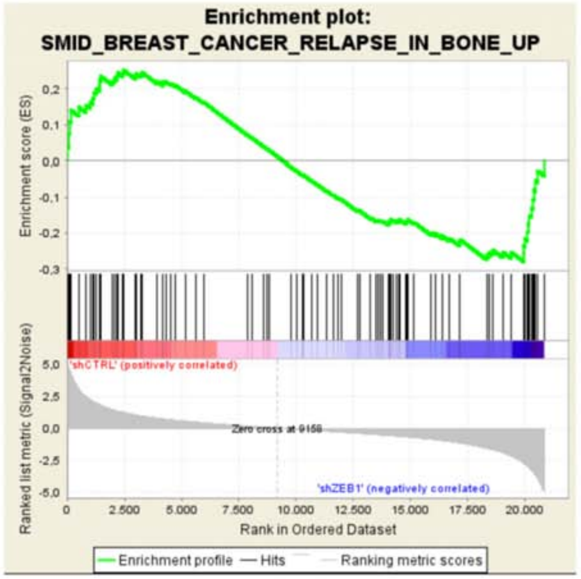

Normalized Enrichment Score (NES): -1.01
Nominal p-value: 0.447 for enrichment in shZEB1

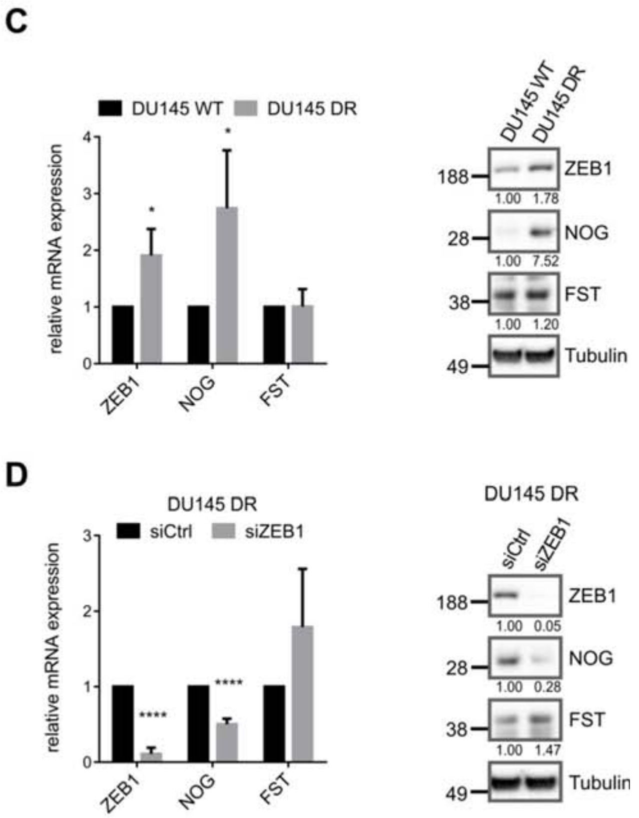

Supplementary Figure S1: ZEB1 regulates BMP-inhibitors but they are not enriched in primary breast tumors. **A.** Expression values (maximum probes) of ZEB1 and the indicated BMP-inhibitors deduced from a whole-genome expression screen comparing shRNA mediated ZEB1 knockdown and control knockdown clones in MDA-MB-231. **B.** Gene-Set-Enrichment-Analysis reveals no significant enrichment of the gene set SMID_BREAST_CANCER_RELAPSE_IN_BONE with the shCtrl or shZEB1 phenotype. **C.** Selection of a docetaxel resistant (DR) subclone from DU145 prostate cancer cells induces ZEB1, as well as NOG and slightly FST expression, when compared to wild type (WT) DU145 as analyzed by qRT-PCR and western blot. qRT-PCR data are represented as mean +SD. **D.** siRNA mediated knockdown of ZEB1 in docetaxel resistant (DR) DU145 results in decreased mRNA and protein expression of NOG relative to siCtrl, as analyzed by qRT-PCR and western blot. FST is regulated antagonistically. qRT-PCR data are represented as mean +SD.

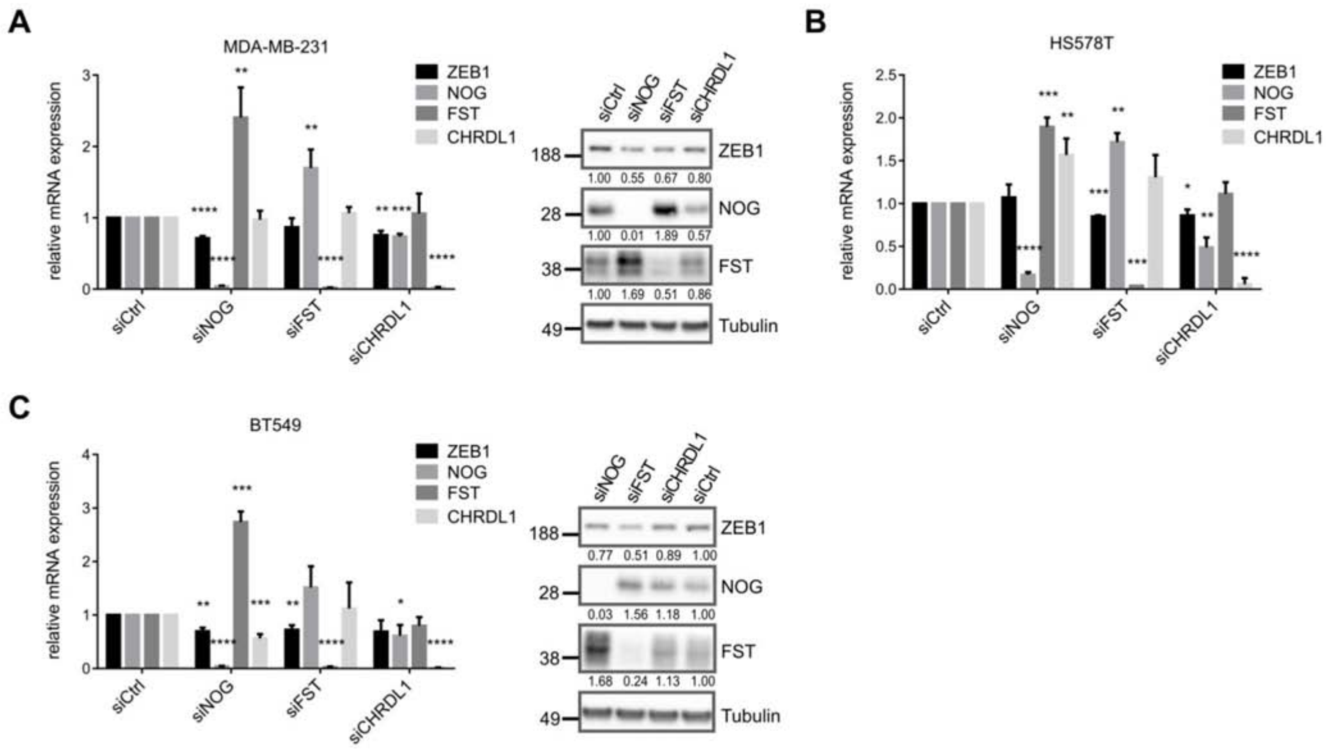

Supplementary Figure S2: BMP-inhibitors NOG and FST are antagonistically regulated in breast cancer cells. A-C. siRNA mediated knockdown of NOG, FST or CHRDL1 in MDA-MB-231, HS578T and BT549 breast cancer cell lines reveals antagonistic regulation of NOG and FST. Depletion of NOG increases FST and vice versa, as analyzed by qRT-PCR and western blot. qRT-PCR data are represented as mean +SD.

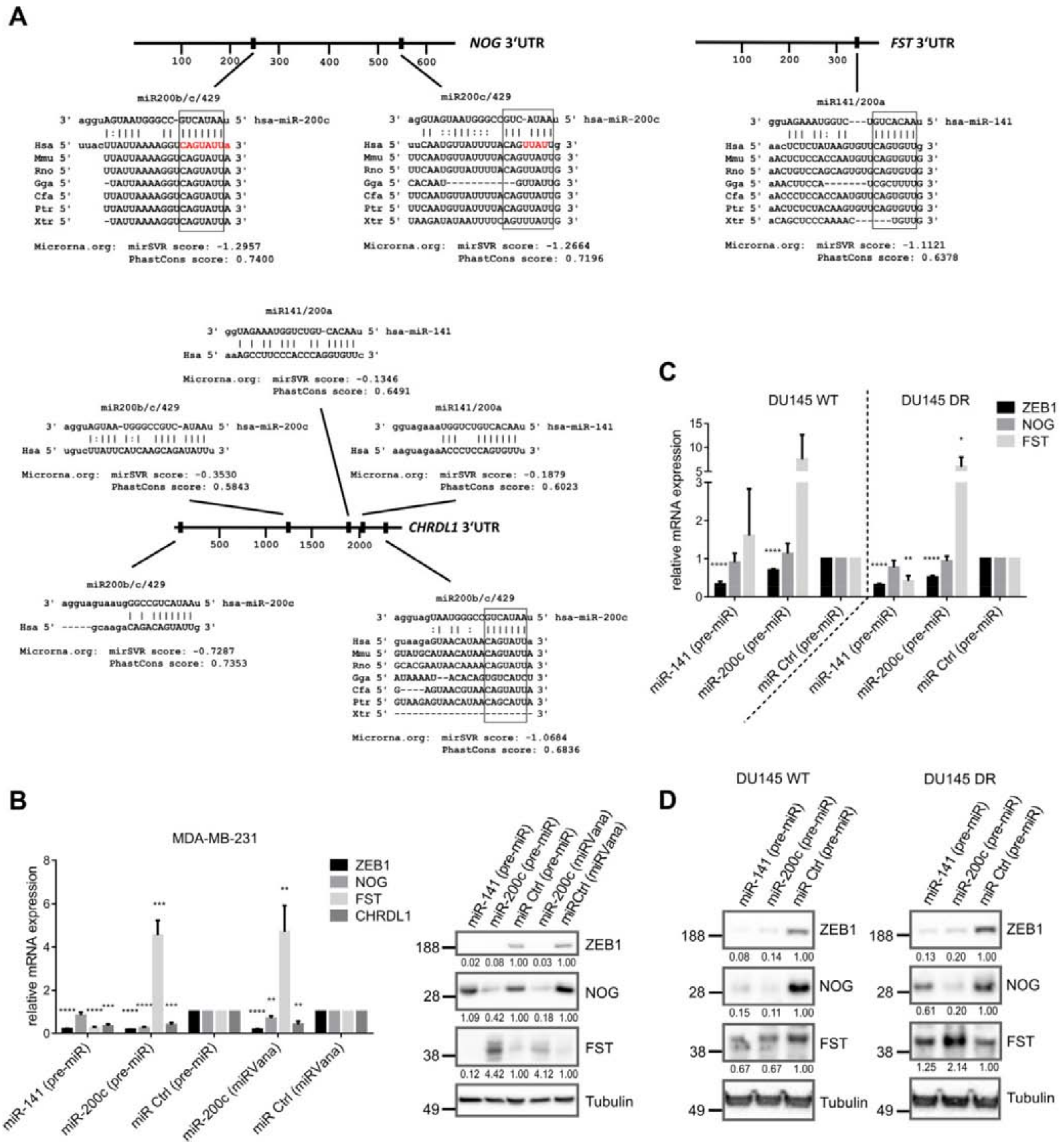

Supplementary Figure S3: BMP-inhibitors are targets of the miR-200 family. A. Schematic representation of the 3′UTRs of human *NOG*, *FST* and *CHRDL1* mRNAs. Predicted binding sites of miR-200 family members are shown with miRSVR scores and interspecies conservation as stated by microRNA.org and targetscan.org. Nucleotides that were deleted by site directed mutagenesis in the NOG 3′UTR-reporter are marked in red. Species abbreviations: Hsa: *Homo sapiens*, Mmu: *Mus musculus*, Rno: *Rattus norwegicus*, Gga: *Gallus gallus*, Cfa: *Canis lupus familiaris*, Ptr: *Pan troglodytes*, Xtr: *Xenopus tropicalis.* **B.** qRT-PCR and western blot analysis show targeting of ZEB1, NOG, FST and CHRDL1 by miR-200c or miR-141 (pre-miR or miRVana from life technologies) in MDA-MB-231. **C, D.** qRT-PCR and western blot analysis show targeting of ZEB1, NOG and FST by miR-200c or miR-141 (pre-miR from life technologies) in wiltype (WT) DU145 prostate cancer cells and docetaxel resistant (DR) DU145.

Supplementary Table S1. Genes positively regulated by ZEB1 are upregulated in breast cancer bone metastases.

NAME	Signal to noise	Rank	*p*-value	Ratio shZEB1/shCtrl
FBN1	1.7422	1	0.001996	0.09
IGFBP4	1.5333	2	0.001996	0.00
BICC1	1.4256	3	0.001996	0.09
MATN3	1.3994	4	0.001996	0.02
FSTL1	1.3916	5	0.001996	0.10
HEG1	1.3575	6	0.001996	0.04
CDH11	1.3321	7	0.001996	0.01
PRSS23	1.3202	8	0.001996	0.07
VIM	1.2278	9	0.001996	0.01
GJA1	1.1864	10	0.001996	0.01
SPOCK1	1.1827	11	0.001996	0.05
NT5E	1.1675	12	0.001996	0.06
FST	1.14	13	0.001996	0.04
COL5A1	1.1127	14	0.001996	0.02
WIPF1	1.0997	15	0.001996	0.06
ASAM	1.09	16	0.001996	0.11
RFTN1	1.0574	17	0.001996	0.08
SLC2A3	1.0545	18	0.001996	0.17
HTRA1	1.0332	19	0.001996	0.04
ZEB1	1.0072	20	0.001996	0.05
FRMD6	0.999	21	0.001996	0.07
SLIT2	0.9894	22	0.001996	0.09
BCAT1	0.9754	23	0.001996	0.01
PTPRM	0.9673	24	0.001996	0.15
GPR68	0.9549	25	0.001996	0.03
HNMT	0.9487	26	0.001996	0.06
EDIL3	0.9284	27	0.001996	0.01
NEDD4	0.9232	28	0.001996	0.15
WNT5B	0.9158	29	0.001996	0.06
FN1	0.9149	30	0.001996	0.06
PTRF	0.913	31	0.001996	0.14
NR3C1	0.9118	32	0.001996	0.10
DOCK4	0.9116	33	0.001996	0.10
NNMT	0.9089	34	0.001996	0.11
LHFP	0.8839	35	0.001996	0.09
HRH1	0.8749	36	0.001996	0.15

(*Continued*)

NAME	Signal to noise	Rank	*p*-value	Ratio shZEB1/shCtrl
CREB3L1	0.8609	37	0.001996	0.09
VEGFC	0.8541	38	0.003992	0.02
SDC2	0.8448	39	0.001996	0.07
PMEPA1	0.8448	40	0.001996	0.10
LPAR1	0.8299	41	0.001996	0.11
ENPP1	0.8216	42	0.001996	0.18
SCCPDH	0.8215	43	0.001996	0.02
BMP2	0.7864	44	0.001996	0.15
MCTP1	0.7746	45	0.001996	0.03
DOCK10	0.7742	46	0.001996	0.06
CSF1	0.7646	47	0.003992	0.06
PTX3	0.758	48	0.003992	0.01
MMP14	0.748	49	0.001996	0.18
PDLIM4	0.7458	50	0.005988	0.05
SNAI2	0.7122	51	0.001996	0.06
LAPTM5	0.707	52	0.001996	0.01
LOC729839	0.6889	53	0.001996	0.15
NOG	0.6807	54	0.001996	0.07
GNG11	0.6434	55	0.003992	0.00
HLA-DRB1	0.6425	56	0.003992	0.09
MLH1	0.6409	57	0.003992	0.14
RAB34	0.6401	58	0.005988	0.01
GGT5	0.6385	59	0.005988	0.05
LY96	0.6327	60	0.001996	0.10
HIC1	0.6318	61	0.00998	0.06
ITGB2	0.6311	62	0.005988	0.10
COL4A1	0.6261	63	0.007984	0.03
CTGF	0.6243	64	0.005988	0.01
CHST11	0.622	65	0.007984	0.11
SRGN	0.6189	66	0.003992	0.00
CD74	0.6171	67	0.007984	0.01
LOX	0.6139	68	0.003992	0.04
SH2B3	0.6088	69	0.005988	0.17
ME3	0.5991	70	0.00998	0.04
TLR4	0.5881	71	0.005988	0.04
HLA-DRA	0.5826	72	0.01198	0.11
HLA-DPA1	0.5784	73	0.00998	0.05
IFI16	0.574	74	0.005988	0.01

(*Continued*)

NAME	Signal to noise	Rank	*p*-value	Ratio shZEB1/shCtrl
G0S2	0.566	75	0.01198	0.01
TTC28	0.556	76	0.01198	0.11
IL6ST	0.5161	77	0.01597	0.17
PRICKLE2	0.5154	78	0.02994	0.14
THBD	0.5014	79	0.01397	0.02
SNAPC1	0.5006	80	0.01996	0.11
SPG20	0.4967	81	0.01796	0.03
CRISPLD1	0.4959	82	0.01597	0.02
GBP3	0.4953	83	0.02395	0.01
HBEGF	0.4901	84	0.01397	0.06
CYR61	0.4815	85	0.01796	0.06
TIMP4	0.4808	86	0.01198	0.06
COL4A2	0.4738	87	0.02794	0.01
JUN	0.4694	88	0.02794	0.12
DPYD	0.4667	89	0.00998	0.03
EFHA2	0.46	90	0.02395	0.03
FOXP1	0.4592	91	0.01397	0.05
TNIP1	0.4588	92	0.01996	0.15
ARMCX1	0.4541	93	0.03992	0.02
ANTXR2	0.4527	94	0.03393	0.02
ARHGDIB	0.4317	95	0.03992	0.03
COL13A1	0.4287	96	0.03393	0.02
FAM26F	0.4264	97	0.0479	0.16
IL31RA	0.4261	98	0.02395	0.04
GALC	0.4206	99	0.03593	0.07
NEGR1	0.4092	100	0.02595	0.03
FMNL1	0.4072	101	0.02395	0.05
RIN2	0.4021	102	0.0499	0.02
APOL3	0.4016	103	0.02595	0.08
ESAM	0.4012	104	0.04591	0.04
PLXDC2	0.3953	105	0.0499	0.08
SLFN12	0.3866	106	0.07984	0.06
PYCARD	0.3824	107	0.0479	0.02
FAM129A	0.3815	108	0.06986	0.07
LRRC33	0.3808	109	0.04591	0.04
GPX3	0.3766	110	0.0499	0.01
EVC	0.3756	111	0.06387	0.02
EDEM1	0.3749	112	0.06387	0.16

(*Continued*)

NAME	Signal to noise	Rank	*p*-value	Ratio shZEB1/shCtrl
LIMCH1	0.3724	113	0.1158	0.10
C8ORF42	0.3701	114	0.07385	0.08
ZNF521	0.3628	115	0.1058	0.11
SHISA2	0.3588	116	0.06986	0.01
CARD6	0.3523	117	0.06587	0.06
DLC1	0.3468	118	0.06786	0.01
ZNF404	0.3335	119	0.1018	0.04
IL6	0.3231	120	0.08782	0.02
GLRB	0.3191	121	0.1198	0.03
LAYN	0.3138	122	0.1257	0.02
ADAMTS1	0.3116	123	0.1257	0.01
NRP1	0.3083	124	0.1257	0.01
IGFBP7	0.3067	125	0.1118	0.00
CCDC8	0.3041	126	0.1417	0.12
WWC3	0.3036	127	0.1377	0.11
ADRB2	0.3034	128	0.1178	0.02
IL1B	0.3017	129	0.1078	0.05
LPHN2	0.2915	130	0.1238	0.03
PSMB8	0.2902	131	0.1557	0.00
KIF7	0.287	132	0.1537	0.02
PEA15	0.2846	133	0.1357	0.17
GNB4	0.2775	134	0.1717	0.01
KIAA0495	0.2745	135	0.1637	0.07
ZNF331	0.2725	136	0.1776	0.15
ROBO1	0.2721	137	0.1597	0.03
RGMB	0.2706	138	0.1756	0.14
GPC6	0.2693	139	0.1856	0.07
LEF1	0.2674	140	0.1796	0.04
MICAL2	0.265	141	0.1876	0.09
VNN1	0.2646	142	0.1856	0.03
FRMD4A	0.2633	143	0.1697	0.07
MEIS3P1	0.2583	144	0.2056	0.06
ROBO4	0.2581	145	0.1896	0.07
NAV3	0.256	146	0.1976	0.04
SPRY1	0.254	147	0.2096	0.16
ECM1	0.2465	148	0.2176	0.09
DUSP10	0.2457	149	0.2236	0.13
SPANXB1	0.244	150	0.2575	0.00

(*Continued*)

NAME	Signal to noise	Rank	*p*-value	Ratio shZEB1/shCtrl
LOC642852	0.2389	151	0.2295	0.17
LOC375295	0.2323	152	0.2315	0.02
DTX3	0.2252	153	0.2535	0.09
SLITRK5	0.223	154	0.2555	0.03
APCDD1L	0.2214	155	0.2375	0.00
LXN	0.2148	156	0.2635	0.04
RIT1	0.2144	157	0.2615	0.13
NEXN	0.2122	158	0.2675	0.03
CAP2	0.21	159	0.2914	0.12
SPANXA1	0.208	160	0.3932	0.00
SLC22A4	0.2077	161	0.2774	0.07
CCDC106	0.2061	162	0.2914	0.07
GNA15	0.2033	163	0.2814	0.02
GBP1	0.2026	164	0.2774	0.01
TFPI2	0.1997	165	0.2415	0.00
MPDZ	0.199	166	0.3054	0.06
PAWR	0.1973	167	0.3553	0.16
CROT	0.1972	168	0.3154	0.09
RAPH1	0.1925	169	0.3553	0.16
TCEAL3	0.1893	170	0.3313	0.01
GPR116	0.1806	171	0.3353	0.01
RARRES3	0.1804	172	0.3373	0.06
CFB	0.1755	173	0.3433	0.07
C6ORF150	0.1736	174	0.3792	0.05
ARSJ	0.1699	175	0.3653	0.04
LRRC34	0.1699	176	0.3673	0.02
LOC339400	0.1685	177	0.3852	0.06
TMEM192	0.1593	178	0.4291	0.11
KAL1	0.1572	179	0.4172	0.04
S100P	0.154	180	0.4152	0.08
NCF2	0.1527	181	0.4431	0.02
C1ORF133	0.1513	182	0.4491	0.05
ZC3H12A	0.1506	183	0.4411	0.17
BASP1	0.1504	184	0.4112	0.00
CNKSR2	0.1441	185	0.477	0.15
LPXN	0.1384	186	0.4551	0.04

(*Continued*)

NAME	Signal to noise	Rank	*p*-value	Ratio shZEB1/shCtrl
CD302	0.1376	187	0.5289	0.03
TCEAL1	0.1289	188	0.495	0.04
NNT	0.1238	189	0.523	0.03
UBR4	0.1226	190	0.5289	0.15
TGFB2	0.1146	191	0.5589	0.03
CBR1	0.1108	192	0.6008	0.01
C4ORF19	0.09379	193	0.6208	0.03
PNMAL1	0.09314	194	0.6447	0.01
RAB32	0.08509	195	0.6587	0.03
CHPT1	0.08491	196	0.6427	0.17
TLR2	0.07477	197	0.6826	0.04
NAV2	0.07387	198	0.7086	0.05
ZNF804A	0.06668	199	0.6387	0.04
C15ORF48	0.06628	200	0.7006	0.00
MAP9	0.06452	201	0.7385	0.07
ACOT1	0.06387	202	0.6866	0.01
PDE10A	0.06318	203	0.7086	0.09
SPANXC	0.06228	204	0.7365	0.00
NLRP3	0.06121	205	0.7425	0.04
CHFR	0.0606	206	0.7405	0.03
LOC142937	0.05893	207	0.7565	0.16
PDCD1LG2	0.05567	208	0.6786	0.13
LOC100130744	0.05229	209	0.7944	0.04
ZSCAN18	0.05196	210	0.7525	0.16
TUSC1	0.04987	211	0.8543	0.02
RPL39L	0.04981	212	0.8303	0.00
EFEMP1	0.04704	213	0.8204	0.01
GPR133	0.04644	214	0.7605	0.10
SLC43A3	0.03955	215	0.8283	0.14
TFE3	0.03574	216	0.8882	0.18
MNS1	0.03451	217	0.8144	0.05
CLCN4	0.03332	218	0.8603	0.09
CD99L2	0.03306	219	0.8523	0.16
C21ORF56	0.02797	220	0.9042	0.06
NRG1	0.02551	221	0.8882	0.02
KCNQ5	0.02133	222	0.8703	0.01

(*Continued*)

NAME	Signal to noise	Rank	*p*-value	Ratio shZEB1/shCtrl
SPTLC3	0.01879	223	0.9182	0.01
C8ORF48	0.01643	224	0.8982	0.05
IFIT3	0.0157	225	0.8743	0.02
TMEM71	0.01405	226	0.8962	0.02
LAMB3	0.01117	227	0.982	0.13
EXT1	0.009627	228	0.9701	0.14
SLFN13	0.00737	229	0.9621	0.11
IGFBPL1	0.005467	230	0.9202	0.01
FAM113B	0.00283	231	0.984	0.05
RGNEF	−0.001106	232	0.998	0.11
ZNF605	−0.001679	233	0.988	0.01
OAS2	−0.002677	234	0.9581	0.11
TRAF1	−0.003147	235	0.9641	0.17
CXCL11	−0.006686	236	0.98	0.06
ACOT12	−0.01205	237	0.9281	0.09
GSTM3	−0.01385	238	0.9681	0.03
APBB1IP	−0.01396	239	0.9661	0.05
TMEM156	−0.01539	240	0.99	0.11
IFIT2	−0.01774	241	0.9701	0.03
ZCCHC18	−0.02323	242	0.9162	0.08
IL7R	−0.02341	243	0.9142	0.03
SERTAD4	−0.02437	244	0.8563	0.06
PRTFDC1	−0.02442	245	0.9062	0.01
A1BG	−0.02916	246	0.8663	0.17
CTSF	−0.03467	247	0.8523	0.16
RSAD2	−0.03638	248	0.9261	0.08
IL8	−0.03668	249	0.8882	0.02
ZNF674	−0.03937	250	0.7864	0.16
ICAM4	−0.04065	251	0.8603	0.07
TOX	−0.04242	252	0.9162	0.10
ZNF439	−0.04386	253	0.8423	0.03
CDH4	−0.04547	254	0.7884	0.03
IRAK2	−0.04656	255	0.8523	0.18
TACSTD2	−0.04711	256	0.8862	0.01
NSAP11	−0.05028	257	0.7864	0.08
MAFF	−0.05451	258	0.7764	0.14

(*Continued*)

NAME	Signal to noise	Rank	*p*-value	Ratio shZEB1/shCtrl
CHRDL1	−0.05506	259	0.8104	0.07
ZC3H12C	−0.06019	260	0.7026	0.16
TAPBPL	−0.06323	261	0.8643	0.09
WNK3	−0.07007	262	0.7226	0.13
RAD54L2	−0.07701	263	0.6806	0.04
TRBC1	−0.07708	264	0.7764	0.08
TUG1	−0.07895	265	0.6707	0.15
HDGFRP3	−0.08136	266	0.6886	0.03
STAP2	−0.0857	267	0.6208	0.11
LOC339290	−0.08873	268	0.6168	0.03
PPAPDC1A	−0.0947	269	0.5968	0.03
LOC285628	−0.1002	270	0.6307	0.01
OASL	−0.1127	271	0.6347	0.10
HDAC9	−0.1128	272	0.6267	0.11
ZNF573	−0.113	273	0.6188	0.14
LY6K	−0.1165	274	0.5768	0.03
CLDN1	−0.119	275	0.5729	0.14
FLJ44054	−0.1248	276	0.5349	0.11
TM4SF1	−0.1414	277	0.4192	0.01
CHIC2	−0.1414	278	0.4291	0.16
CXORF61	−0.1416	279	0.519	0.07
PCDHGA4	−0.1494	280	0.4251	0.05
KCNMB3	−0.1548	281	0.5309	0.03
C6ORF155	−0.1602	282	0.4411	0.01
ZNF470	−0.1603	283	0.513	0.16
HOXD3	−0.1615	284	0.4631	0.08
TUSC3	−0.171	285	0.4012	0.01
KCNN3	−0.1728	286	0.3573	0.01
C3	−0.1758	287	0.3713	0.03
NACAP1	−0.1841	288	0.3693	0.17
ACSL5	−0.1852	289	0.4731	0.14
CSF2	−0.1893	290	0.3473	0.03
PLCB4	−0.1932	291	0.3313	0.17
FGF5	−0.1963	292	0.2874	0.04
WT1	−0.1964	293	0.4371	0.02
KISS1	−0.2031	294	0.2974	0.08
STAC	−0.213	295	0.3194	0.11
KRT7	−0.2142	296	0.2455	0.02

(*Continued*)

NAME	Signal to noise	Rank	*p*-value	Ratio shZEB1/shCtrl
LOC728705	−0.2154	297	0.3832	0.15
QPCT	−0.2185	298	0.3273	0.03
GCKR	−0.2317	299	0.2655	0.10
PLAC8	−0.2341	300	0.3473	0.01
SPRYD5	−0.2442	301	0.2335	0.13
GAS2	−0.2492	302	0.3792	0.07
NRSN2	−0.2566	303	0.1936	0.02
HOMER2	−0.2599	304	0.2116	0.09
QSOX1	−0.2667	305	0.1816	0.10
C18ORF18	−0.2733	306	0.1677	0.10
CRIM1	−0.2745	307	0.1796	0.12
FOXA2	−0.276	308	0.4531	0.04
ZNF426	−0.2828	309	0.1537	0.02
NXF5	−0.2843	310	0.1637	0.16
ZNF432	−0.285	311	0.1796	0.07
ATXN7	−0.2874	312	0.1537	0.13
ZNF529	−0.2902	313	0.2076	0.07
ANKRD1	−0.2914	314	0.2016	0.03
VLDLR	−0.2988	315	0.1776	0.08
STC1	−0.2998	316	0.1457	0.06
ZNF788	−0.3101	317	0.1717	0.01
EGFR	−0.3124	318	0.1198	0.14
ZNF569	−0.3232	319	0.1277	0.03
ZNF71	−0.3348	320	0.1018	0.06
TSPYL5	−0.3355	321	0.09581	0.04
OGFRL1	−0.3406	322	0.0998	0.11
CHST10	−0.3441	323	0.09381	0.09
CLDN11	−0.3456	324	0.1138	0.09
TMEM163	−0.3459	325	0.1497	0.04
IL1RAPL1	−0.3467	326	0.1098	0.13
MYEF2	−0.3469	327	0.06188	0.05
LOC100130938	−0.3476	328	0.09381	0.10
SMAP1	−0.3612	329	0.06786	0.07
MANSC1	−0.3745	330	0.05389	0.18
SNAP91	−0.3795	331	0.07784	0.04
ZNF420	−0.3835	332	0.06587	0.06

(*Continued*)

NAME	Signal to noise	Rank	*p*-value	Ratio shZEB1/shCtrl
IGFBP1	−0.3928	333	0.06587	0.01
GCNT2	−0.3983	334	0.03792	0.01
SPAG16	−0.4483	335	0.01597	0.05
ZNF615	−0.4586	336	0.03792	0.17
TIMM17B	−0.477	337	0.02196	0.16
ZNF140	−0.4777	338	0.02395	0.04
LOC643650	−0.4844	339	0.02595	0.11
KRT81	−0.4917	340	0.01597	0.00
ZNF20	−0.5487	341	0.01597	0.30
SHC3	−0.5591	342	0.001996	0.13
USP44	−0.5638	343	0.005988	0.02
STK40	−0.58	344	0.00998	0.09
CCNA1	−0.6185	345	0.001996	0.02
FOXF2	−0.6476	346	0.005988	0.14
C21ORF7	−0.6566	347	0.005988	0.10
ZNF260	−0.7255	348	0.001996	0.04
GFAP	−0.7562	349	0.001996	0.11
SLAIN1	−1.2514	350	0.001996	0.04

The top 350 genes positively regulated by ZEB1 were ranked according to differential expression (signal to noise ratio) in bone metastases vs. other metastatic sites by GENE-E program. 110 out of 350 genes are significantly higher expressed in bone metastases with $p < 0.05$. Column 5 shows the ratio mean shZEB1/mean shCtrl calculated from the expression array data of MDA-MB-231 with stable shRNA mediated knockdown of ZEB1.

Supplementary Table S2. Sequences of all oligonucleotides, siRNAs, miRNAs and AntagomiRs

Oligonucleotides

**Primer ChIP**	
NOG for	5′-TACCTGAGGCAGAAGGTGCT-3′
NOG rev	5′-CTGACTGGTCCGTTTTGGTT-3′
FST −1500bp for	5′-AGGTCACCCGCTTTTACCAG-3′
FST −1500bp rev	5′-GAAAGTGGGCATGAATCACA-3′
FST +50bp for	5′-AATTGGGAAGGGTTTCCAGA-3′
FST +50bp rev	5′-GGGAGCTTTCAAAAGTTCAGTG-3′
CHRDL1 for	5′-GGGGAGTCAGAGGGTAGTCG-3′
CHRDL1 rev	5′-CTCTGCACTGGATGCACCT-3′
EPCAM for	5′-GCCAGGTAAAAGCTCAAAGG-3′
EPCAM rev	5′-GCGGGAACTGGATAGAGGA-3′
miR-200c for	5′-CAGGAGGACACACCTGTGC-3′
miR-200c rev	5′-TCCCCTGGTGGCCTTTAC-3′
HPRT1 for	5′-TGAGAGTTCAAGTTGAGTTTGGA-3′
HPRT1 rev	5′-TGATAATTTTACTGGCGATGTCA-3′
**Primer qRT-PCR**	
ACTB for	5′-GCCCTGAGGCACTCTTCCA-3′
ACTB rev	5′-TTGCGGATGTCCACGTCA-3′
ZEB1 for	5′-AAGAATTCACAGTGGAGAGAAGCCA-3′
ZEB1 rev	5′-CGTTTCTTGCAGTTTGGGCATT-3′
NOG for	5′-ATTCTGCCCCGTGCTGTA-3′
NOG rev	5′-TGCCCACCTTCACGTAGC-3′
FST for	5′-TGCCACCTGAGAAAGGCTAC-3′
FST rev	5′-TGGATATCTTCACAGGACTTTGC-3′
CHRDL1 for	5′-GCCGAGTCAGATGTCCAAAT-3′
CHRDL1 rev	5′-ACTGGGGGTAAGGAGTCTGG-3′
MMP9 (mouse) for	5′-ACGACATAGACGGCATCCA-3′
MMP9 (mouse) rev	5′-GCTGTGGTTCAGTTGTGGTG-3′
ACTB (mouse) for	5′-TTACGGATGTCAACGTCA-3′
ACTB (mouse) rev	5′-GCCCTGAGGCTCTTTTCCA-3′
**Primer cloning promoters**	
NOGpromoter long for (XhoI)	5′-ATAATCTCGAGCACAGAAAGGTAGGGCTGGA-3′
NOGpromoter short for (XhoI)	5′-ATAATCTCGAGTAGGGGCTGTGTGTGTGTGT-3′
NOGpromoter long/short rev (HindIII)	5′-ATAATAAGCTTCTGGCTGAGCTGGTTCTAGG-3′

(*Continued*)
